# Effect of Pressures and Durations of Cupping Therapy on Skin Blood Flow Responses

**DOI:** 10.3389/fbioe.2020.608509

**Published:** 2020-12-08

**Authors:** Xiaoling Wang, Xueyan Zhang, Jeannette Elliott, Fuyuan Liao, Jing Tao, Yih-Kuen Jan

**Affiliations:** ^1^College of Rehabilitation Medicine, Fujian University of Traditional Chinese Medicine, Fuzhou, China; ^2^Rehabilitation Engineering Lab, Department of Kinesiology and Community Health, University of Illinois at Urbana-Champaign, Champaign, IL, United States; ^3^Disability Resources and Educational Service, University of Illinois at Urbana-Champaign, Champaign, IL, United States; ^4^Department of Biomedical Engineering, Xi'an Technological University, Xi'an, China

**Keywords:** cupping therapy, dose response, laser Doppler, skin blood flow, reactive hyperemia

## Abstract

Cupping therapy has been widely used in treating musculoskeletal impairments. However, there is no specific guideline on selecting the intensity of cupping therapy, including the pressure and duration. The objective of this study was to investigate the effect of different pressures and durations of cupping therapy on skin blood flow responses. A 2 × 2 factorial design, including two negative pressures at −225 and −300 mmHg and two durations at 5 and 10 min, was tested in 12 healthy participants. The four protocols of cupping therapy were tested in four different days. Skin blood flow was measured using laser Doppler flowmetry on the left triceps (the SJ12 acupoint). Skin blood flow after cupping therapy was expressed as a ratio of skin blood flow before cupping therapy. The results showed that −300 mmHg caused a significant increase in peak skin blood flow (16.7 ± 2.6 times) compared to −225 mmHg (11.1 ± 2.2 times, *p* < 0.05) under 5-min duration. The largest difference in skin blood flow is between −300 mmHg for 5 min (16.7 ± 2.6 times) and −225 mmHg for 10 min (8.1 ± 2.3 times, *p* < 0.01). Our findings demonstrated that a higher value (300 mmHg) of negative pressure is more effective on increasing skin blood flow compared to a lower value (225 mmHg). Also, a shorter duration (5 min) causes a larger peak and total skin blood flow compared to a longer duration (10 min). This study provides the first evidence showing the effect of pressures and durations of cupping therapy on skin blood flow responses.

## Introduction

Cupping therapy is widely used to manage musculoskeletal diseases in China and Egypt (Aboushanab and Alsanad, 2018), including neck pain (Kim et al., 2018), lower back pain (Huang et al., 2013), myofascial pain syndrome (Charles et al., 2019), osteoarthritis (Li et al., 2017), and pain in athletes (Bridgett et al., 2018). Recently, elite athletes, such as Michael Phelps and Russel Westbrook, revealed these circular marks on their shoulders through the media (Chirali, 2014). Subsequently, the awareness and curiosity about cupping therapy has been continuing to grow in the western countries. However, there is insufficient clinical guideline on selecting the intensity of cupping therapy (Mehta and Dhapte, 2015; Aboushanab and Alsanad, 2018; Jan et al., 2020).

The pressure and duration factors of cupping therapy are two main factors to change the intensity of cupping therapy and may influence effectiveness of cupping therapy (Lowe, 2017). The current principle on adjusting the intensity of cupping therapy is that if the absolute value of negative pressure is too low, cupping therapy may not be effective. On the other hand, if the absolute value of negative pressure is too large, cupping therapy would cause discomfort and pain to the patient. Although different intensities of cupping therapy are believed to cause various effects on treating symptoms (Cui and Cui, 2012), there are only few studies in the literature to guide the selection of the pressure and duration of cupping therapy (Lowe, 2017; Aboushanab and Alsanad, 2018).

Although there is no consensus about the potential mechanisms of cupping therapy, a local increase in blood flow is mostly accepted as a critical factor (Sloas et al., 2016; Lowe, 2017; Hou et al., 2020). After the removal of cup, cupping therapy causes an increase in local blood flow and lymphatic flow that may be helpful for relieving myofascial pain and improving soft tissue healing (Jafri, 2014; Sloas et al., 2016; Lowe, 2017). The study of the response of skin blood flow (SBF) to various intensities of cupping therapy could be used to assess the efficacy of various intensities of cupping therapy (Lowe, 2017). In this study, the efficacy refers to the ability of cupping therapy to achieve the desired effect of increasing blood flow. Tian showed that SBF significantly increased after cupping therapy at three negative pressures [i.e., −225 mmHg (−0.03 MPa), −300 mmHg (−0.04 MPa), and −375 mmHg (−0.05 MPa)] for 5 min. The author indicated that the absolute value of negative pressure should not exceed −375 mmHg (−0.05 MPa), which may be harmful to the tissues. For example, negative pressure at −450 mmHg (−0.06 MPa) is considered too large and may harm the soft tissue within the cupping cup. The author also indicated that the absolute value of negative pressure should be above −225 mmHg (−0.03 MPa) for increasing SBF after cupping therapy. For example, negative pressure at −150 mmHg (−0.02 MPa) is too small and may not benefit the treated area (Tian et al., 2007).

Regarding the duration factor of cupping therapy, it is advised that the duration should be within 10 min to avoid the blister (Zhou et al., 2014). Liu et al. demonstrated that SBF increased after the application of negative pressure at −300 mmHg (−0.04 MPa) for 10 min in healthy volunteers (Liu et al., 2014). Tian et al. also showed that SBF significantly increased after negative pressure at −225 mmHg (−0.03 MPa) for 5 min (Tian et al., 2007). Based on these studies, the duration between 5 and 10 min has been recommended in cupping therapy. However, there is no specific guideline on choosing a duration between 5 and 10 min. Overall, it remains largely unknown about the interactions between different pressures and durations of cupping therapy on SBF responses.

In this study, we proposed to study the effect of various pressures and durations on SBF responses. The results can be used to better understand the effect of the intensity of cupping therapy on SBF. Specifically, a 2 × 2 factorial design was used to examine the effect of negative pressures at −225 and −300 mmHg and durations at 5 and 10 min on SBF responses. To the best of our knowledge, this is the first study to investigate the effect of pressure and duration of cupping therapy on SBF responses.

## Methods

A 2 × 2 factorial, including two negative pressures at −225 and −300 mmHg and two durations at 5 and 10 min, repeated measure design was used in this study. A total of four protocols of cupping therapy was tested, including (A) −225 mmHg for 5 min, (B) −225 mmHg for 10 min, (C) −300 mmHg for 5 min, and (D) −300 mmHg for 10 min. A counterbalanced design was used to minimize the order effect. The specific test orders of four protocols of cupping therapy in all participants are shown in Table 1.

**Table 1:** The counterbalanced order of four different cupping therapy protocols used in this study, including −225 mmHg × 5 min, (B) −225 mmHg × 10 min, (C) −300 mmHg × 5 min, and (D) −300 mmHg × 10 min.

**Subject**	**The order of four protocols of cupping therapy**
#1	A, B, C, D
#2	B, A, C, D
#3	A, B, D, C
#4	B, A, D, C
#5	C, D, A, B
#6	D, C, A, B
#7	C, D, B, A
#8	D, C, B, A
#9	A, D, B, C
#10	B, C, A, D
#11	A, D, C, B
#12	B, C, D, A

The selection of negative pressure of cupping therapy between −225 and −300 mmHg for this study was based on the commonly used setting (Tian et al., 2007; Huber et al., 2011). We did not test the negative pressure between −300 and −375 mmHg because these higher pressures caused discomfort in our preliminary studies. The selection of duration of cupping therapy between 5 and 10 min for this study was also based on clinical practice (Liu et al., 2014; Sucher, 2019). The four protocols of cupping therapy would cover most intensities of cupping therapy. The site for cupping therapy was chosen at the Xiaoluo acupoint (SJ12) of the left arm (i.e., non-dominant arm). The selection of the non-dominant arm was to avoid the influence of cupping therapy on the dominant arm required to perform activities of daily living in research participants. SJ12 is at the triceps and is used to relieve the pain of shoulder and upper limb (Mo Qian et al., 2012).

The inclusion criteria were healthy adults aged between 18 and 40 years and were fine with marks on the triceps after cupping therapy. The participants were told that these marks would subside in several days. The exclusion criteria were as follows: no allergy, nodule, and swelling on the cupping skin; abnormal blood pressure; and abnormal temperature. The target population of participants was the students of the University of Illinois. All of the recruited participants signed an informed consent before being enrolled in this study. The protocol was approved by the University of Illinois at Urbana-Champaign Institutional Review Board (#20334).

Laser Doppler flowmetry (LDF) (PeriFlux 5000, Perimed, Ardmore, PA) with a thermostatic probe (Probe 455, 23-mm in diameter, Perimed) was used to measure SBF. The LDF device delivers a low-power beam (2 mW) of a solid-state diode laser source (780 nm wavelength) to the skin and measures backscattered light in the tissue caused by moving red blood cells. According to the Doppler principle, some of the frequency of the backscattered light changes and some does not. Based on the portions of frequency changes, the number of moving red blood cells and the velocity of these cells can be used to estimate SBF (i.e., skin perfusion) (Rajan et al., 2009). SBF signals were sampled at 32 Hz. Peak SBF, total SBF, and recovery time of the SBF response to cupping therapy were used to characterize SBF response to cupping therapy (Hagisawa et al., 1994; Jan et al., 2005, 2013; Jan, 2020). An electric cupping device was used to apply negative pressure (Powerpress Pro, Neomedic, Chatsworth, CA). The use of an electric cupping device allowed the authors to apply the standard pressure over four protocols of cupping therapy in all research participants (Huber et al., 2011). The cup with the 45-mm diameter (the inner diameter as 45 mm and the outer diameter as 53 mm) was used to apply cupping therapy. The rim width of the cup is 8 mm in total (each side of the rim as 4 mm) and is not sharp, so as not to cause pain in the participant. This size is commonly used in clinical practice.

The room temperature was maintained at 24 ± 2°C throughout the experiment. All participants were required to accustom themselves to the room temperature for 30 min before the data collection. Baseline SBF level of each participant was tested in the relaxed, supine position with the elbow in full extension, forearm in full supination, and wrist in the neutral position. SBF was continuously measured using laser Doppler probe for 10 min to establish the baseline value. Then, cupping therapy at one of four protocols was applied on the center of SJ12 acupoint. The specific pressure and duration of cupping therapy was pre-determined and randomly assigned to the participant (Table 1). After cupping therapy (removal of the cup), the laser Doppler probe was again placed on the SJ12 for measuring SBF responses for 10 min. The second protocol of cupping therapy was applied after 2–4 days to prevent carryover effort of a previous protocol of cupping therapy. Then, the third and fourth protocols were conducted followed the same procedures. The participants were required to visit the lab in four different days to complete this study.

**Figure 1:**
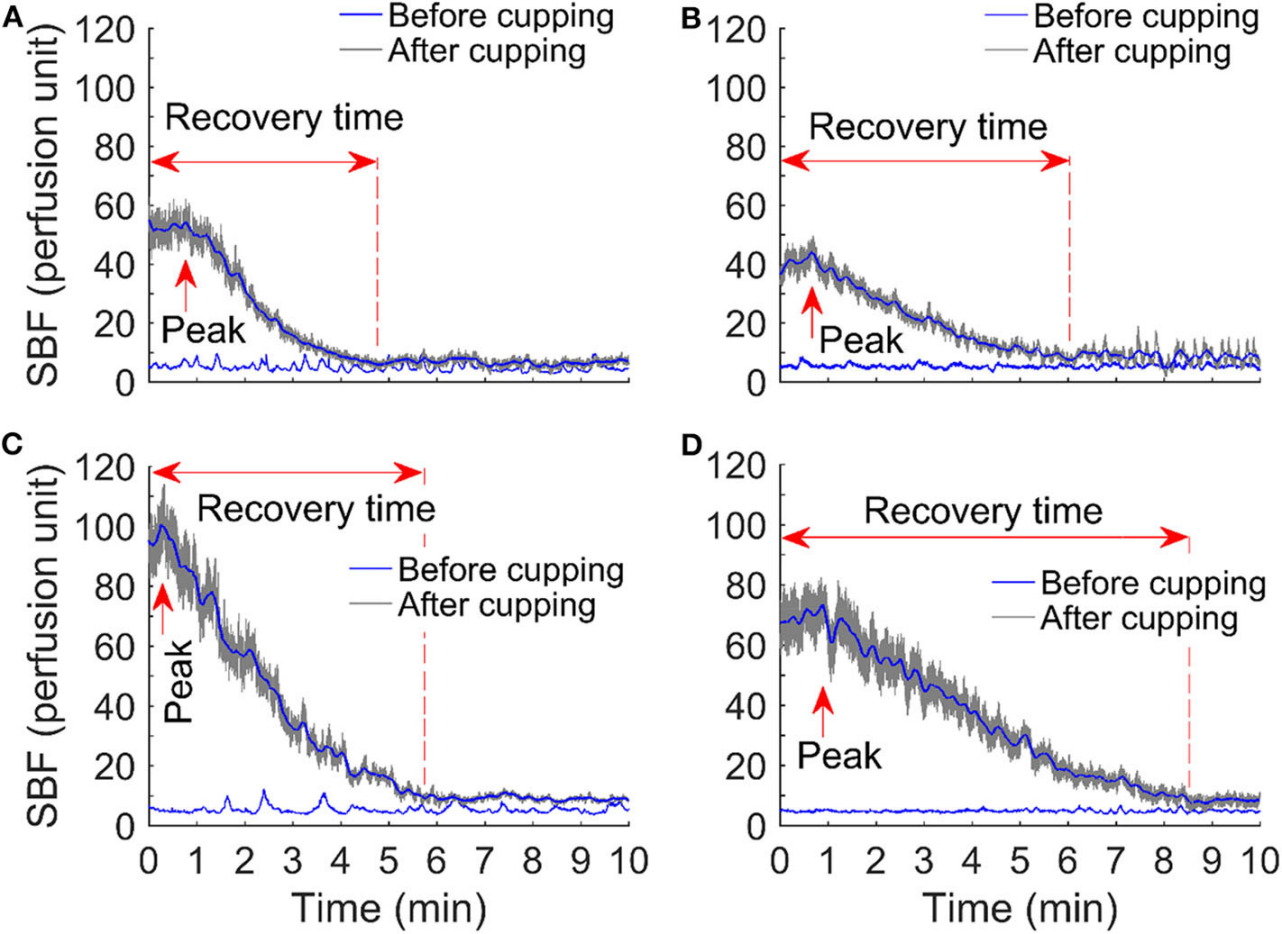
Comparison of skin blood flow responses before and after cupping therapy in four protocols: **(A)** −225 mmHg × 5 min, **(B)** −225 mmHg × 10 min, **(C)** −300 mmHg × 5 min, and **(D)** −300 mmHg × 10 min. Among all skin blood flow responses, an initial, rapid increase in skin blood flow is observed; the peak value of skin blood flow reaches in about 1–3 min. Peak skin blood flow is about 5–20 times compared to the baseline skin blood flow.

SBF responses after cupping therapy were quantified as peak SBF (in perfusion unit), total SBF (in perfusion unit), and recovery time (in seconds) (Figure 1) (Jan et al., 2013; Jan, 2020). Peak SBF was defined as the maximum value of blood flow after cupping therapy. Total SBF was defined as the blood flow value integrated over the hyperemia period after cupping therapy. The recovery time was defined as the SBF value reached to a steady level. SBF values after cupping therapy were expressed as a ratio of baseline SBF (no unit) to overcome temporal variations of SBF measured in four different days (Jan et al., 2005, 2013; Jan, 2020). A two-way analysis of variance (ANOVA) with repeated measures was used to assess the differences in SBF response between and within groups. The Wilcoxon Signed Rank test was used to examine the significant difference between two protocols. The significance level was set at *p* < 0.05. All statistical analyses were performed using SPSS 26.

## Results

Twelve participants who met the inclusion and exclusion criteria were recruited into the study from the students of University of Illinois. Their average age (SD) was 29.5 ± 8.5 years. The body mass index (BMI) was 22.3 ± 2.6 kg/m^2^. Their average (SD) systolic blood pressure, diastolic blood pressure, and heart rate was 106.6 ± 15.4 mmHg, 72.3 ± 7.6 mmHg, and 75.1 ± 7.9 beats/min, respectively.

Figure 1 shows examples of the SBF responses after four protocols of cupping therapy, including (A) −225 mmHg for 5 min, (B) −225 mmHg for 10 min, (C) −300 mmHg for 5 min, and (D) −300 mmHg for 10 min. Among all SBF responses, an initial, rapid increase in SBF is observed; the peak value of SBF reaches in about 1–3 min. Peak SBF is about 5–20 times compared to the baseline SBF.

**Figure 2:**
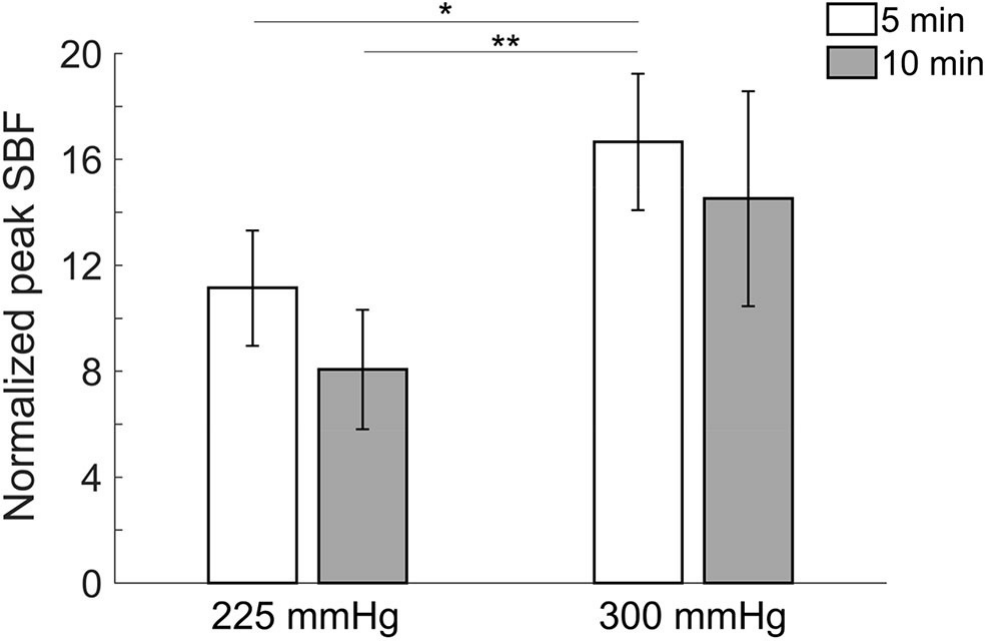
Comparisons of normalized peak skin blood flow in four protocols. All pressures refer to the absolute value of negative pressure of cupping therapy. Under the duration of 5 min, −300 mmHg causes a significant increase in peak skin blood flow (16.7 ± 2.6 times) compared to −225 mmHg (11.1 ± 2.2 times, *p* < 0.01). A significant difference is observed between −300 mmHg for 5 min (16.7 ± 2.6 times) and −225 mmHg for 10 min (8.1 ± 2.3 times, *p* < 0.01). Values are mean ± SE. SBF, skin blood flow. *p < 0.05 and **p < 0.01.

Comparisons of normalized peak SBF after four protocols of cupping therapy are shown in Figure 2. Under the duration of 5 min, −300 mmHg causes a significant increase in peak SBF (16.7 ± 2.6 times) compared to −225 mmHg (11.1 ± 2.2 times, *p* < 0.01). Under the duration of 10 min, −300 mmHg shows a trend of causing higher peak SBF (14.5 ± 4.1 times) compared to −225 mmHg (8.1 ± 2.3 times, 0.05 < *p* < 0.1). When comparing SBF after the same pressure at −225 mmHg, the 5-min cupping therapy causes higher peak SBF (11.1 ± 2.2 times) compared to a 10-min cupping therapy (8.1 ± 2.3 times, 0.05 < *p* < 0.1); while, there is no difference between 5-min (16.7 ± 2.6 times) and 10-min (14.5 ± 4.1 times) after the same pressure at −300 mmHg. A significant difference is observed between −300 mmHg for 5 min (16.7 ± 2.6 times) and −225 mmHg for 10 min (8.1 ± 2.3 times, *p* < 0.01).

**Figure 3:**
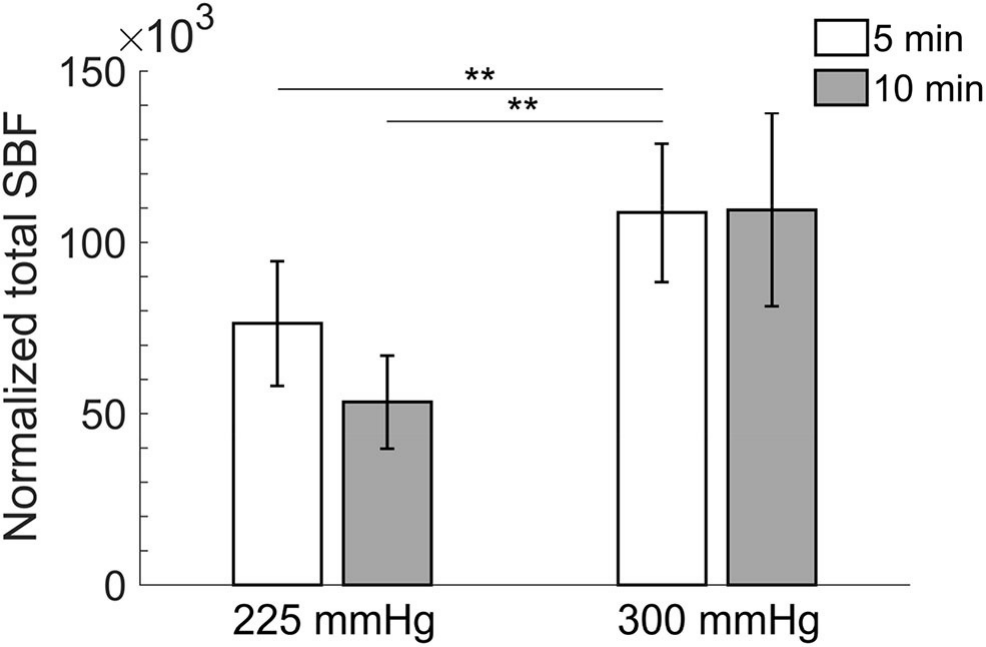
Comparisons of normalized total skin blood flow in four protocols. All pressures refer to the absolute value of negative pressure of cupping therapy. Under the duration of 5 min, −300 mmHg causes a significant increase in total skin blood flow [(108.7 ± 20.2) × 10^3^ times] compared to −225 mmHg [(76.4 ± 18.2) × 10^3^ times, *p* < 0.01]. The largest difference in total skin blood flow is between −300 mmHg for 5 min [(108.7 ± 20.2) × 10^3^ times] and −225 mmHg for 10 min [(53.4 ± 13.6) × 10^3^ times, *p* < 0.01]. Values are mean ± SE. SBF, skin blood flow. **p < 0.01.

Comparisons of normalized total SBF after the four protocols of cupping therapy are shown in Figure 3. Under the duration of 5 min, −300 mmHg causes a significant increase in total SBF [(108.7 ± 20.2) × 10^3^ times] compared to −225 mmHg [(76.4 ± 18.2) × 10^3^ times, *p* < 0.01] after cupping therapy. Under the duration of 10 min, −300 mmHg shows a trend of causing more total SBF [(109.5 ± 28.1) × 10^3^ times] compared to −225 mmHg [(53.4 ± 13.6) × 10^3^ times, 0.05 < *p* < 0.1] after cupping therapy. Under the same pressure at −225 mmHg, the 5-min cupping therapy shows a trend of causing more total SBF [(76.4 ± 18.2) × 10^3^ times] compared to 10-min cupping therapy [(53.4 ± 13.6) × 10^3^ times, 0.05 < *p* < 0.1]; while, there is no significant difference between 5 and 10 min under the same pressure at −300 mmHg. The largest difference in total SBF is between −300 mmHg for 5 min [(108.7 ± 20.2) × 10^3^ times] and −225 mmHg for 10 min [(53.4 ± 13.6) × 10^3^ times, *p* < 0.01].

**Figure 4:**
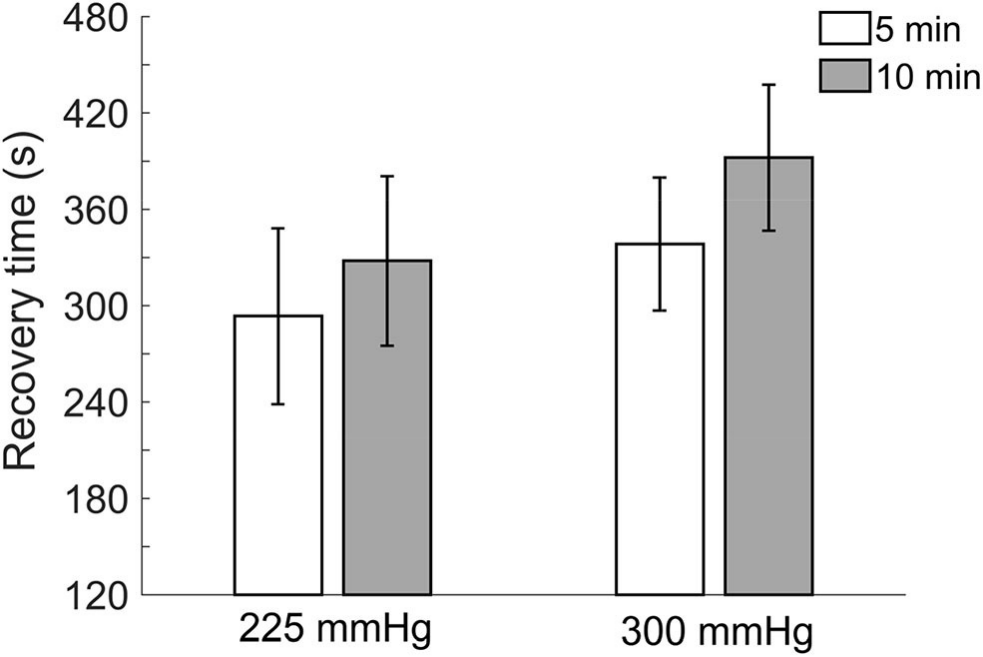
Comparisons of recovery time in four protocols. All pressures refer to the absolute value of negative pressure of cupping therapy. There is a trend that recovery time of 10-min cupping therapy is longer than 5-min cupping therapy when under the same pressure. Values are mean ± SE. SBF, skin blood flow.

Comparisons of recovery time after the four protocols of cupping therapy are shown in Figure 4. There is a trend that recovery time of 10-min cupping therapy is longer than 5-min cupping therapy when under the same pressure. Under −225 mmHg pressure, recovery time after 5-min cupping therapy (293.6 ± 54.8 s) appears shorter than 10-min cupping therapy (328.0 ± 52.8 s, non-significant). Under −300 mmHg pressure, recovery time after 5-min cupping therapy (338.4.6 ± 41.5 s) appears shorter than 10-min cupping therapy (392.2 ± 45.4 s, non-significant).

## Discussion

This study provides the first evidence showing the effect of pressures and durations of cupping therapy on SBF responses. Under different combinations of pressures and durations of cupping therapy, cupping therapy can cause different effects on the microvascular system. Our finding indicates a need to determine the dose response of cupping therapy on SBF responses, especially the pressure and duration factors. In this study, we further confirm that under the same duration of cupping therapy, a higher absolute value (−300 mmHg) of negative pressure is more effective on increasing SBF compared to a lower absolute value (−225 mmHg) of negative pressure after cupping therapy. Also, under the same negative pressure, a shorter duration (5 min) may cause a larger peak and total SBF compared to a longer duration (10 min) after cupping therapy.

Using the non-invasive, optical device, LDF, the SBF response to four different intensities of cupping therapy were quantified in this study for the first time. As shown in Figure 1, such blood flow responses (i.e., a rapid increase to reach the peak value and then a gradual decrease) are similar to the reactive hyperemic response caused by an ischemic stress on the soft tissue. Reactive hyperemia has been used as a clinical tool to assess blood flow function in various patient populations and the intensity of applied mechanical stress (Wong et al., 2003). The response of reactive hyperemia is associated with the degree of ischemia. In this study, the ischemia was caused by the mechanical interactions between the cupping cup and negative pressure of cupping therapy (Rendell and Wells, 1998; Cunningham et al., 2002; Tham et al., 2006). During cupping therapy, soft tissues within the cup will be sucked away from the bone and are under tensile stress, and soft tissues directly under the rim of the cup are under the compressive stress. Under this condition, blood flow cannot enter or leave the vasculature of soft tissues inside the cup. For a duration of 5 min at −225 mmHg, the applied negative pressure causes soft tissue ischemia. When the cup was removed, a rapid increase in SBF occurred inside the area of the cup; and the pattern of SBF increases is dependent on the intensity of cupping therapy. This is consistent with the previous studies showing an increase in SBF after cupping therapy (Tian et al., 2007; Kim et al., 2011; Liu et al., 2014). In this study, we further confirmed that a reactive hyperemic pattern occurs after cupping therapy. Furthermore, these SBF responses can be quantified and characterized by peak SBF, total SBF, and recovery time. In the literature, mean SBF is used to assess cupping therapy on SBF that may not fully reveal the physiological regulations. The potential mechanism of reactive hyperemia is the myogenic control mediated by local vascular smooth muscle cells of the blood vessels (Wong et al., 2003; Jan et al., 2013; Liao et al., 2013). The result from this study indirectly support a local regulation in response to cupping therapy through demonstrating reactive hyperemic responses after cupping therapy (Figure 1). To the best of our knowledge, this is the first evidence documenting the reactive hyperemic responses after various intensities of cupping therapy.

The degree of reactive hyperemia after cupping therapy may be associated with the extent of ischemia caused during the application of cupping therapy (Daniel et al., 1981; Wong et al., 2003; Jan et al., 2013). Peak SBF of reactive hyperemia is considered an indicator of the shear force of blood flow acting on the vascular wall (Henrion, 2005; Jan et al., 2013). The four protocols of cupping therapy in this study caused different degrees of ischemia of soft tissue under the cupping cup that would subsequently causes different magnitudes of shear forces after the removal of the cupping cup. In this study, we demonstrated that a higher absolute value of negative pressure (−300 mmHg) of cupping therapy can cause more increases in blood flow (El Sayed et al., 2013). Our findings indicate that larger absolute values of negative pressure should be used when the pressure is within the safe range. The larger absolute value of negative pressure causes more increases in the fluid filtration of intravascular vessels to interstitial spaces (Rendell and Wells, 1998; Cunningham et al., 2002; Tham et al., 2006). This observation is also consistent with the fact that a larger absolute value of negative pressure may cause a larger hyperemic response until reaching the threshold (Daniel et al., 1981; Wong et al., 2003; Jan et al., 2013). More studies are needed to determine the safe range of cupping pressure for effectively increasing SBF.

Regarding the duration of cupping therapy, our results showed a trend that 5-min cupping therapy increased more SBF than 10-min cupping therapy. This indicates that the combination of the magnitude and duration of cupping therapy produces different shear forces for eliciting different blood flow responses. Thus, the duration factor should also be considered in practice of cupping therapy. After cupping therapy, more tissue fluid and leakage of blood flow were inside the cup until an equilibrium point. The increase in SBF would reach a stable level when fluid and blood flow cannot increase anymore (El Sayed et al., 2013). A typical response of a ruptured capillary is an increase in blood flow occurring within a few seconds. With the longer duration, the accumulation of vasodilators inside soft tissue may be gradually dissipated and inactive. Thus, 5-min cupping therapy may be more effective on increasing SBF then 10-min cupping therapy. Zhao et al. demonstrated that skin color of after cupping therapy (ecchymosis) was darker after 10 min than 30 and 20 min (Zhao et al., 2009). Although the skin color of ecchymosis is related to many factors, SBF is considered a major factor. According to these findings, the duration of cupping therapy should not exceed 10 min although a duration of more than 10 min is commonly used (Zhao et al., 2009).

Recovery time shows a trend with longer recovery time when the absolute value of negative pressure was larger and duration was longer. The recovery time is also related to degree of ischemia of soft issue; a larger ischemic stress needs a longer time for ischemic tissues to remove metabolic wastes and restore SBF (Hagisawa et al., 1994; Rossi et al., 2007). However, these differences are not statistical significance. Among three outcome measurements, recovery time appears not as sensitive as peak and total SBF responses.

There are limitations of this study. First, we examined the effect of cupping therapy on the triceps. Our results may not be generalized to different muscles with different characteristics (e.g., thickness). Also, the site for the application of cupping therapy was the SJ12 acupoint in this study. It remains largely unknown whether the application of cupping therapy at the non-acupoint site would produce similar results. Further studies should explore the influence of acupoint on the SBF responses. Second, this study was conducted in a homogenous group of participants with similar BMIs. The results may not be generalized to groups of overweight and obesity. Third, the authors measure skin blood responses using LDF. Future studies may use other devices to measure muscle blood flow responses after cupping therapy.

## Conclusion

This study provides the first evidence showing the effect of pressures and durations of cupping therapy on SBF responses. Under different combinations of pressures and durations of cupping therapy, cupping therapy can cause different blood flow responses that are related to the effectiveness of cupping therapy. Our finding indicates a need to determine the dose response of cupping therapy on SBF responses, especially the pressure and duration factors.

## Data Availability Statement

The original contributions presented in the study are included in the article/supplementary material, further inquiries can be directed to the corresponding author/s.

## Ethics Statement

The studies involving human participants were reviewed and approved by University of Illinois at Urbana-Champaign. The patients/participants provided their written informed consent to participate in this study.

## Author Contributions

XW, XZ, and Y-KJ designed the study, collected the data, and analyzed the data. XW, XZ, JE, FL, JT, and Y-KJ interpreted the data and prepared the manuscript.

## Conflict of Interest

The authors declare that the research was conducted in the absence of any commercial or financial relationships that could be construed as a potential conflict of interest.

## References

[B1] Aboushanab T. S. Alsanad S. (2018). Cupping therapy: an overview from a modern medicine perspective. J. Acupunct. Meridian. Stud. 11, 83–87. 10.1016/j.jams.2018.02.001 29436369

[B2] Bridgett R. Klose P. Duffield R. Mydock S. Lauche R. (2018). Effects of cupping therapy in amateur and professional athletes: systematic review of randomized controlled trials. J. Altern. Complement. Med. 24, 208–219. 10.1089/acm.2017.0191 29185802

[B3] Charles D. Hudgins T. Macnaughton J. Newman E. Tan J. Wigger M. (2019). A systematic review of manual therapy techniques, dry cupping and dry needling in the reduction of myofascial pain and myofascial trigger points. J. Bodyw. Mov. Ther. 23, 539–546. 10.1016/j.jbmt.2019.04.001 31563367

[B4] Chirali I. Z. (2014). Traditional Chinese medicine cupping therapy. Oxford Churchill Livingston, 1–16. 10.1016/B978-0-7020-4352-9.00011-4

[B5] Cui S. Cui J. (2012). [Progress of researches on the mechanism of cupping therapy]. Zhen Ci Yan Jiu 37, 506–510. 23383463

[B6] Cunningham D. D. Henning T. P. Shain E. B. Young D. F. Hannig J. Barua E.. (2002). Blood extraction from lancet wounds using vacuum combined with skin stretching. J. Appl. Physiol. 92, 1089–1096. 10.1152/japplphysiol.00798.2001 11842044

[B7] Daniel R. K. Priest D. L. Wheatley D. C. (1981). Etiologic factors in pressure sores: an experimental model. Arch. Phys. Med. Rehabil. 62, 492–498. 7305643

[B8] El Sayed S. M. Mahmoud H. S. Nabo N. M. (2013). Methods of wet cupping therapy (Al-Hijamah): in light of modernmedicine and prophetic medicine. Altern. Integr. Med. 2, 1–16. 10.4172/2327-5162.1000111

[B9] Hagisawa S. Ferguson-Pell M. Cardi M. Miller S. D. (1994). Assessment of skin blood content and oxygenation in spinal cord injured subjects during reactive hyperemia. J. Rehabil. Res. Dev. 31, 1–14. 8035356

[B10] Henrion D. (2005). Pressure and flow-dependent tone in resistance arteries. Role of myogenic tone. Arch. Mal. Coeur. Vaiss. 98, 913–921. 16231579

[B11] Hou X. He X. Zhang X. Liao F. Hung Y. J. Jan Y. K. (2020). Using laser Doppler flowmetry with wavelet analysis to study skin blood flow regulations after cupping therapy. Skin Res. Technol. 10.1111/srt.12970. [Epub ahead of print]. 33089947

[B12] Huang C. Y. Choong M. Y. Li T. S. (2013). Effectiveness of cupping therapy for low back pain: a systematic review. Acupunct. Med. 31, 336–337. 10.1136/acupmed-2013-010385 23886511

[B13] Huber R. Emerich M. Braeunig M. (2011). Cupping - is it reproducible? Experiments about factors determining the vacuum. Complement. Ther. Med. 19, 78–83. 10.1016/j.ctim.2010.12.006 21549258

[B14] Jafri M. S. (2014). Mechanisms of myofascial pain. Int. Sch. Res. Not. 2014:523924. 10.1155/2014/523924 25574501 PMC4285362

[B15] Jan Y. K. (2020). The effects of local cooling rates on perfusion of sacral skin under externally applied pressure in people with spinal cord injury: an exploratory study. Spinal Cord 58, 476–483. 10.1038/s41393-019-0378-x 31700147

[B16] Jan Y. K. Brienza D. M. Geyer M. J. (2005). Analysis of week-to-week variability in skin blood flow measurements using wavelet transforms. Clin. Physiol. Funct. Imaging 25, 253–262. 10.1111/j.1475-097X.2005.00621.x 16117727

[B17] Jan Y. K. Hou X. He X. Guo C. Jain S. Bleakney A. (2020). Using elastographic ultrasound to assess the effect of cupping size of cupping therapy on stiffness of triceps muscle. Am. J. Phys. Med. Rehabil. 10.1097/PHM.0000000000001625. [Epub ahead of print]. 33065576

[B18] Jan Y. K. Liao F. Rice L. A. Woods J. A. (2013). Using reactive hyperemia to assess the efficacy of local cooling on reducing sacral skin ischemia under surface pressure in people with spinal cord injury: a preliminary report. Arch. Phys. Med. Rehabil. 94, 1982–1989. 10.1016/j.apmr.2013.03.022 23583346

[B19] Kim J. I. Kim T. H. Lee M. S. Kang J. W. Kim K. H. Choi J. Y.. (2011). Evaluation of wet-cupping therapy for persistent non-specific low back pain: a randomised, waiting-list controlled, open-label, parallel-group pilot trial. Trials 12:146. 10.1186/1745-6215-12-146 21663617 PMC3141528

[B20] Kim S. Lee S. H. Kim M. R. Kim E. J. Hwang D. S. Lee J.. (2018). Is cupping therapy effective in patients with neck pain? A systematic review and meta-analysis. BMJ Open 8:e021070. 10.1136/bmjopen-2017-021070 30397006 PMC6231582

[B21] Li J. Q. Guo W. Sun Z. G. Huang Q. S. Lee E. Y. Wang Y.. (2017). Cupping therapy for treating knee osteoarthritis: the evidence from systematic review and meta-analysis. Complement. Ther. Clin. Pract. 28, 152–160. 10.1016/j.ctcp.2017.06.003 28779923

[B22] Liao F. Y. Burns S. Jan Y. K. (2013). Skin blood flow dynamics and its role in pressure ulcers. J. Tissue Viability 22, 25–36. 10.1016/j.jtv.2013.03.001 23602509 PMC3658615

[B23] Liu W. Piao S.-A. Meng W.-X. Wei L.-H. (2014). Effects of cupping on blood flow under skin of back in healthy human. World J. Acupunct. Moxibustion 23, 50–52. 10.1016/S1003-5257(13)60061-6

[B24] Lowe D. T. (2017). Cupping therapy: an analysis of the effects of suction on skin and the possible influence on human health. Complement. Ther. Clin. Pract. 29, 162–168. 10.1016/j.ctcp.2017.09.008 29122256

[B25] Mehta P. Dhapte V. (2015). Cupping therapy: a prudent remedy for a plethora of medical ailments. J. Tradit. Complement. Med. 5, 127–134. 10.1016/j.jtcme.2014.11.036 26151023 PMC4488563

[B26] Mo Qian M. X.-J. Feng-Long W. Yuan-Shi L. Yang T. (2012). Dialectical applieation of long round needle to treat with stroke after hemiplegia shoulderhand stage I syndrome. J. Cervicodynia Lumbodynia 33, 3211–3214.

[B27] Rajan V. Varghese B. Van Leeuwen T. G. Steenbergen W. (2009). Review of methodological developments in laser Doppler flowmetry. Lasers Med. Sci. 24, 269–283. 10.1007/s10103-007-0524-0 18236103

[B28] Rendell M. S. Wells J. M. (1998). Ischemic and pressure-induced hyperemia: a comparison. Arch. Phys. Med. Rehabil. 79, 1451–1455. 10.1016/S0003-9993(98)90243-X 9821909

[B29] Rossi M. Carpi A. Di Maria C. Franzoni F. Galetta F. Santoro G. (2007). Post-ischaemic peak flow and myogenic flowmotion component are independent variables for skin post-ischaemic reactive hyperaemia in healthy subjects. Microvasc. Res. 74, 9–14. 10.1016/j.mvr.2007.02.006 17399744

[B30] Sloas D. C. Stewart S. A. Sweat R. S. Doggett T. M. Alves N. G. Breslin J. W.. (2016). Estimation of the pressure drop required for lymph flow through initial lymphatic networks. Lymphat. Res. Biol. 14, 62–69. 10.1089/lrb.2015.0039 27267167 PMC4926202

[B31] Sucher B. M. (2019). Suction decompression of the carpal tunnel. J. Am. Osteopath. Assoc. 119, 464–468. 10.7556/jaoa.2019.083 31233112

[B32] Tham L. M. Lee H. P. Lu C. (2006). Cupping: from a biomechanical perspective. J. Biomech. 39, 2183–2193. 10.1016/j.jbiomech.2005.06.027 16126216

[B33] Tian Y. Qin L. Zhang W. (2007). Preliminary observation of different negative pressure of cupping therapy on skin blood flow. Acupunct. Res. 32, 184–185.

[B34] Wong B. J. Wilkins B. W. Holowatz L. A. Minson C. T. (2003). Nitric oxide synthase inhibition does not alter the reactive hyperemic response in the cutaneous circulation. J. Appl. Physiol. 95, 504–510. 10.1152/japplphysiol.00254.2003 12692141

[B35] Zhao X. X. Tong B. Y. Wang X. X. Sun G. L. (2009). [Effect of time and pressure factors on the cupping mark color]. Zhongguo Zhen Jiu. 29, 385–388. 19489497

[B36] Zhou X. Ruan J. W. Xing B. F. (2014). [Analysis on the adverse events of cupping therapy in the application]. Zhongguo Zhen Jiu. 34, 1023–1025. 25543441

